# Tomato Ripeness Detection and Localization Based on the Intelligent Inspection Robot Platform

**DOI:** 10.3390/s26103174

**Published:** 2026-05-17

**Authors:** Xinrui Li, Long Liang, Yubo Liu, Jingxia Lu

**Affiliations:** 1 College of Smart Agriculture, Nanjing Agricultural University, Nanjing 211800, China; li--xinrui@stu.njau.edu.cn (X.L.); lliang@stu.njau.edu.cn (L.L.); 2 The Bartlett Faculty of the Built Environment, University College London, London WC1E 6BT, UK

**Keywords:** tomato ripeness detection, intelligent inspection robot, YOLOv8n, UWB, lightweight design, precision agriculture

## Abstract

The field inspection and ripeness detection of tomatoes in China remain heavily dependent on manual labor, while existing robotic solutions often exhibit limited functionality, poor environmental adaptability, prohibitive hardware costs, and unstable positioning accuracy. To address these limitations, this study proposes an intelligent tomato inspection robot that seamlessly integrates real-time ripeness recognition with precise spatial localization. Built upon a Raspberry Pi 5 core controller, the robot employs a lightweight, layered modular architecture designed to flexibly navigate complex agricultural environments. A comprehensive, multi-dimensional image dataset of tomato ripeness was constructed to train a three-category detection model based on the YOLOv8n architecture. Following 413 training epochs, the model demonstrated exceptional performance, achieving an overall mAP@0.5 of 87.8% and an mAP@0.5:0.95 of 72.7% on the held-out test dataset. In field inspections, the system achieved detection precisions of 82.22% for immature tomatoes, 92.66% for half-ripened tomatoes, and 100% for fully ripe tomatoes, successfully identifying all ripe tomatoes and satisfying the practical demands of field inspection. Furthermore, the integration of an Ultra-Wideband positioning system yielded an overall Root Mean Square Error of 0.231 m, successfully confining positioning errors to within 0.24 m to fully satisfy the stringent localization demands of crop-level inspection. Field evaluations confirmed that under optimal configurations, the robot can efficiently inspect a 50-m planting row in 10 min (±1 min) and maintains a continuous operational battery life of 2 h (±10 min). The core contribution of this work is the system-level integration and optimization of technologies for greenhouse agriculture. This integrated design achieves low hardware cost and high deployment flexibility, addressing longstanding challenges of labor-intensive inspection and delayed harvesting, and delivering a practical solution for intelligent tomato plantation management.

## 1. Introduction

Amid the rapid growth and rising productivity of China’s tomato industry, major producing regions increasingly demand “timely harvesting, precise management, and reduced manual inspection.” At the same time, facility agriculture is continuously transitioning towards automation, digitization, networking, and intelligence. Modern technologies are finding growing application in facility agriculture, making research and innovation in agricultural robots a core direction of precision agriculture [1]. Furthermore, deep learning technology has been widely adopted in precision agriculture, providing efficient solutions for tasks such as crop phenotype analysis, pest and disease detection, and maturity recognition [2,3]. Consistent with this trend, solutions integrating deep learning-based intelligent perception with robotic inspection have emerged as a key direction in modern tomato cultivation.

Currently, researchers have extensively investigated machine learning methods for tomato phenotype detection. The YOLO-CT model proposed by Qin et al. [4], which incorporates an SA attention mechanism and a high-resolution small object prediction head, achieves precise detection and localization of multiple cherry tomato varieties and their picking points. It exhibits strong generalization and is suitable for embedded devices, but it lacks autonomous mobility and is not suited to large-scale field inspection scenarios. Meng et al. [5] improved the YOLOv7-tiny + DeepSORT algorithm by integrating depth information mapping with a SimAM attention module. This enabled accurate detection, tracking, and counting of cherry tomatoes at multiple maturity stages. This solved issues of background interference and cross-frame matching, yet their approach still lacks autonomous mobility and relies on fixed-track inspection.

To address these limitations, researchers are continuously advancing intelligent inspection robot technologies. The four-wheel-steering orchard robot proposed by Raikwar et al. [6] uses a model-based design to achieve autonomous navigation in GPS-denied environments. It offers high navigation stability, with a steering offset error of only 0.2–0.4 degrees. However, its diesel-electric hybrid hardware architecture results in high costs. Moreover, in scenarios with dense vegetation cover like orchards, it is prone to signal occlusion, leading to a GNSS-denied state and consequently positioning interruption or a sharp drop in accuracy. The retrofitted robot presented by Ospina et al. [7] utilizes RTK-GNSS + GIS to achieve multi-machine coordination and automatic speed regulation, significantly reducing operational errors. Nevertheless, RTK-GNSS is more susceptible to signal occlusion than UWB and requires base stations for differential correction, adding to system complexity. The AG-LOAM framework proposed by Teng et al. [8], which is mounted on a Clearpath Jackal robot, achieves precise navigation and mapping in unstructured agricultural environments using a single LiDAR. It offers strong localization robustness and adapts well to complex terrain. Although LiDAR-based systems offer high localization accuracy, their widespread adoption in agricultural robotics is constrained by high acquisition and operational costs, sensor complexity, and data processing demands, especially for smallholder farming scenarios [9]. In contrast, UWB technology has been widely adopted in agricultural robot localization due to its centimeter-level accuracy, strong interference immunity, and low power consumption, while maintaining significantly lower hardware costs compared to LiDAR [10]. Therefore, this study adopts a vision-based camera for tomato maturity detection and a UWB-based positioning system, achieving a cost-effective yet precise solution for field inspection.

All three aforementioned robotic platforms suffer from high hardware costs and lack integrated crop detection functionality. Meanwhile, most existing agricultural inspection robots focus solely on optimizing either navigation or detection performance, failing to provide a complete closed-loop framework that integrates target detection, spatial positioning, and decision feedback [11]. To address these gaps, this paper presents a cost-effective, high-performance inspection robot that integrates crop detection, autonomous mobility, and precise positioning for tomato maturity monitoring and ripe fruit localization. The robot features a lightweight, compact design with low overall hardware expenditure. It employs a Raspberry Pi 5 as the host computer and is equipped with a front-mounted two degrees of freedom (2-DOF) camera gimbal, enabling autonomous movement without reliance on fixed tracks. For maturity detection, this study adopts the YOLOv8n model for tomato phenotype analysis, which demonstrates reliable performance for tomato ripeness detection across the entire growth cycle. This model offers high recognition accuracy, low computational overhead, and easy deployment. Furthermore, the robot integrates a UWB positioning system that provides centimeter-level accuracy, strong anti-interference capability, simple base station setup, and low hardware cost.

The remainder of this paper is organized as follows. Section 2 details the design of the tomato inspection robot. Section 3 describes the dataset construction and YOLOv8n detection model development. Section 4 introduces the UWB positioning system design. Section 5 presents the field experiments and results. And Section 6 summarizes the conclusions and future research directions.

## 2. Materials and Methods

### 2.1. Overall Design

The tomato intelligent inspection robot consists of a core control module, a 2-DOF camera gimbal (YAHBOOM, Shenzhen, China) module, a motion module, and a support module. It is controlled by a Raspberry Pi 5 (Raspberry Pi Holdings plc, Cambridge, UK), which connects to an expansion board for expanded functionality. With four-wheel drive and differential wheel control, the robot can turn and navigate around obstacles, ensuring stable and efficient inspection on rugged terrain.

### 2.2. Mechanical Structure and Functions

**Figure 1:**
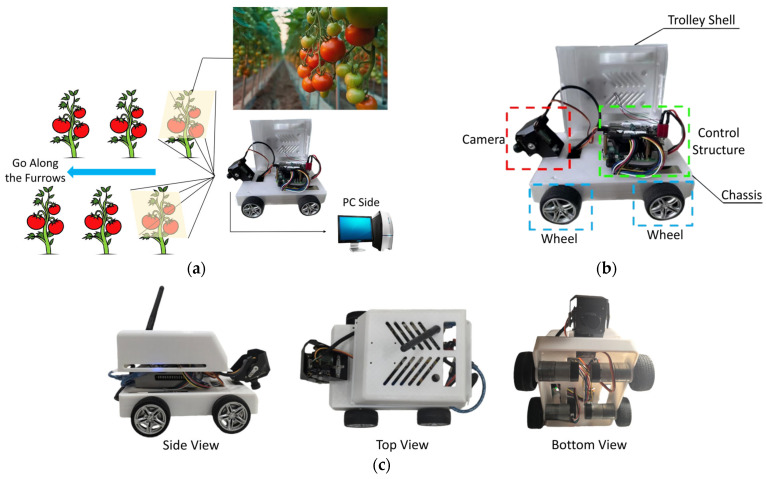
Overall structure of the tomato intelligent inspection robot. (**a**) The robot’s basic workflow; (**b**) The robot’s overall structure; (**c**) The side, top, and bottom views of the robot.

The tomato intelligent inspection robot uses a Raspberry Pi 5 as its host computer, connects to an ESP32-S3 expansion board (YAHBOOM, Shenzhen, China), runs on Robot Operating System 2 (ROS2), and is equipped with a UWB tag for localization. It can move along furrows in a tomato plantation, inspecting plants on either side, as shown in Figure 1a. The robot uses the Raspberry Pi 5 as its central processing and scheduling unit, which offers low cost and high flexibility [12]. It is also equipped with a 2-DOF camera gimbal. Through this gimbal, the robot uses the YOLOv8n model to detect and recognize tomatoes; combined with a BP-TWR-30 UWB positioning module (BlueDot Infinite Technology, Shenzhen, China), it determines the locations of ripe tomatoes and promptly alerts workers for harvesting. Figure 1b shows the overall structure of the robot, while Figure 1c shows its side, top, and bottom views.

**Figure 2:**
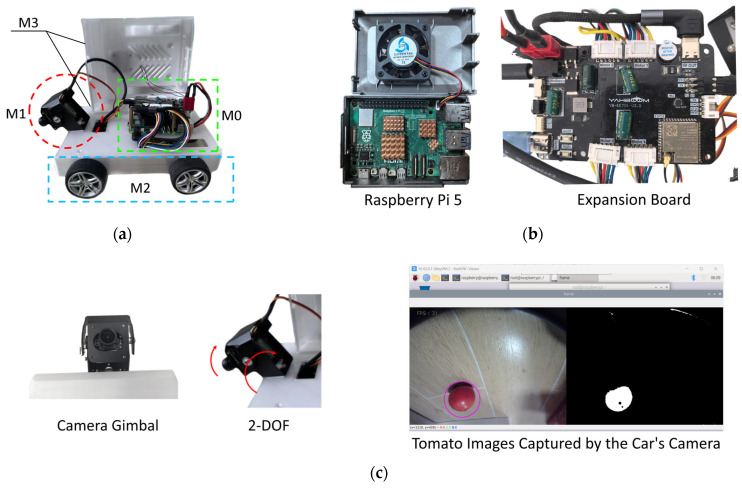

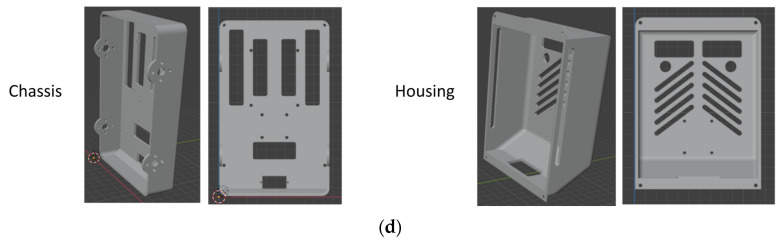
Modular design of the robot. (**a**) The robot’s four modules; (**b**) The robot’s core control layer; (**c**) The robot’s camera module; (**d**) The robot’s support layer.

The intelligent tomato inspection robot has a total weight of only 0.55 kg and dimensions of 34 cm × 18 cm × 14 cm. Owing to its lightweight and compact design, it can maneuver flexibly between tomato planting rows, adapting well to the inspection requirements of narrow field operational environments. The robot adopts a layered modular hardware architecture consisting of four main layers: Core Control (M0), Data Acquisition (M1), Motion (M2), and Support (M3), as shown in Figure 2a. Each layer exchanges data and commands through standardized hardware interfaces. The Core Control Layer serves as the computing and scheduling core, responsible for controlling robot movement, adjusting camera posture, and coordinating all functional modules. The Data Acquisition Layer captures multi-angle tomato images and acquires centimeter-level field localization information. The Motion Layer executes precise inspection path control based on commands from the Core Control Layer. The Support Layer houses all hardware modules and ensures the robot’s adaptability when traversing field furrows. These layers communicate bidirectionally via interfaces such as CSI and UART, with an overall hardware data transmission delay of less than 20 ms, which meets the requirements for real-time field inspection. The main components of the robot are listed in Table 1.

The core of the Core Control Layer (M0) is the Raspberry Pi 5, which integrates a Broadcom BCM2712 quad-core 2.4 GHz processor and 8 GB of LPDDR4X memory. It delivers substantially higher computing performance than mainstream microcontrollers such as STM32 and Arduino, while reducing hardware costs by approximately 60% compared to the Jetson Nano. Additionally, it is equipped with an active cooler that ensures less than 5% performance degradation during 2 h of continuous operation, making it well-adapted to the high-temperature environments characteristic of agricultural greenhouses. The Raspberry Pi series of development boards has been widely validated as a preferred solution for the core control units of agricultural Internet of Things (IoT) devices [3,13].

Complementing the host computer, the ESP32-S3 expansion board serves as the low-level controller of the inspection robot. It receives and parses motion commands transmitted from the Raspberry Pi 5 via the UART interface, enabling precise closed-loop control of the four-wheel differential drive motors and the servos of the 2-degree-of-freedom (2-DOF) camera gimbal. Furthermore, the expansion board is pre-flashed with microROS firmware, which facilitates seamless interoperability with the Robot Operating System 2 (ROS2) environment running on the Raspberry Pi 5. The physical assembly of the Raspberry Pi 5 and the ESP32-S3 expansion board is illustrated in Figure 2b.

RGB camera-based monocular vision systems represent a cost-effective and highly interoperable solution for crop phenotypic data acquisition. Furthermore, multi-view data acquisition facilitated by a multi-degree-of-freedom (DOF) gimbal is indispensable for achieving high-precision crop detection [14]. The camera gimbal integrated into the Data Acquisition Layer (M1) features two degrees of freedom, offering a 90° vertical rotation range and a 180° horizontal rotation range. This design maximizes the visual coverage of tomato plants in inter-row spaces, enabling efficient acquisition of representative sample data across diverse orientations and vertical heights. Figure 2c illustrates the robot’s camera module and representative tomato images captured and processed by the module, with detection results visualized via image binarization.

For the Motion Layer (M2), the robot employs a differential wheel drive configuration. By independently adjusting the rotational speeds of the left and right wheels, the robot can perform smooth turning maneuvers and obstacle avoidance, enabling robust adaptation to uneven field terrains. The two-wheel differential drive is a well-established locomotion scheme for mobile robots, whose core advantage lies in the seamless integration of steering and propulsion through independent speed control of individual wheels. Its kinematic properties and steering stability have been extensively validated in prior research [15]. Turning to the Support Layer (M3), the robot’s protective housing is fabricated via 3D printing using acrylonitrile butadiene styrene (ABS) engineering plastic—a material renowned for its excellent mechanical strength, high toughness, and low weight [16]. The 3D structural design of the housing is presented in Figure 2d.

The tomato intelligent inspection robot can perform various movements, including forward, backward, turning, and obstacle avoidance. In drive mode, the wheels enable rapid movement on flat surfaces and allow quick switching to a slow inspection mode as needed. In inspection mode, the robot can move slowly or come to a stop, using its 2-DOF camera gimbal for detection.

## 3. Dataset Processing and YOLO Model Design

### 3.1. Tomato Data Acquisition and Preprocessing

#### 3.1.1. Source Image Data Acquisition

The tomato intelligent inspection robot features a camera gimbal system, which provides ample video data for multi-category tomato detection, tracking, and localization. After filtering the acquired video data, numerous images were extracted. These images were then annotated and compiled into a dataset for subsequent training of the multi-category tomato detection model. The specific workflow is illustrated in Figure 1a.

#### 3.1.2. Tomato Image Data Processing

Tomatoes were categorized into three classes: immature tomatoes, half-ripened tomatoes, and fully ripened tomatoes. Frames were extracted from videos captured by the robot, yielding a total of 2097 tomato images with varying resolutions and angles. The three classes were approximately evenly distributed in a ratio of 1:1:1.

**Figure 3:**
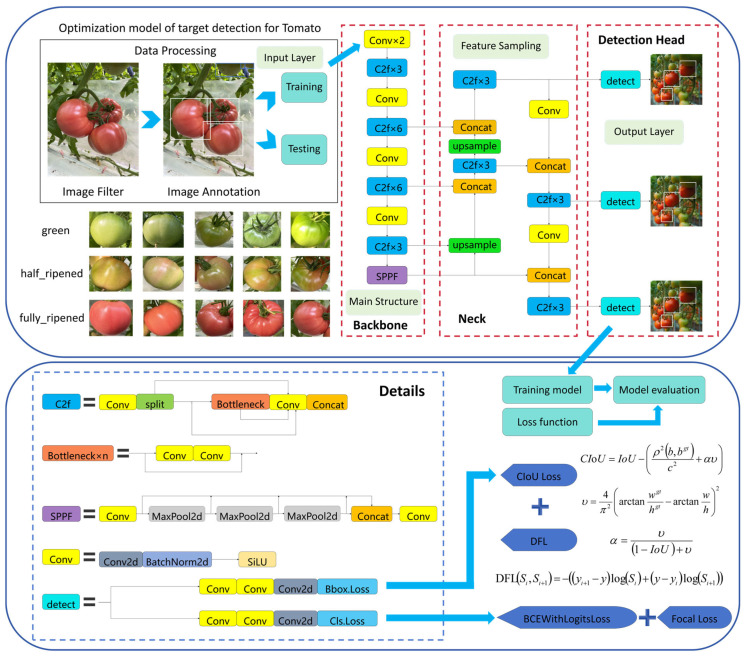
The network architecture and details of YOLOv8.

Prior to YOLOv8n model training, we performed data annotation using the open-source tool LabelImg. The three tomato categories were annotated as follows: Category 0 for immature tomatoes (labeled “green”), Category 1 for half-ripened tomatoes (labeled “half_ripened”), and Category 2 for fully ripened tomatoes (labeled “fully_ripened”). Following annotation, a dataset in YOLOv8 format was generated, with annotation filenames matching the corresponding image filenames. We extracted representative images of each tomato category from the collected dataset to create a sample gallery. The annotation process and a subset of this sample gallery are shown in Figure 3. The dataset was partitioned into training, validation, and test sets in an 8:1:1 ratio based on planting rows. All images captured from the same planting row were assigned to exactly one subset, ensuring strict separation between the training and evaluation sets (validation and test) at the planting row level. This partitioning strategy eliminated data leakage caused by adjacent images from the same row appearing in multiple subsets.

### 3.2. YOLOv8 Tomato Multi-Category Detection Model

After completing data acquisition, dataset creation, and platform construction, this section details how tomato images are processed and analyzed using the YOLOv8n model. Convolutional Neural Networks, including the YOLO series, are among the most efficient machine learning models for crop vision detection [13]. This study adopts the YOLOv8n model, a lightweight variant of YOLOv8, and fine-tunes it for tomato maturity detection.

#### 3.2.1. Model Network Structure

Before designing the tomato multi-category joint learning detection model, the aforementioned dataset was constructed. In this section, the YOLOv8 model is used to train on this dataset. The network architecture and key details of YOLOv8 are illustrated in Figure 3. YOLOv8 offers five variants for multi-category object detection, which differ primarily in network depth. Among these, YOLOv8n is the shallowest. Furthermore, we compared four mainstream lightweight YOLO models (YOLOv5n, YOLOv7-tiny, YOLOv8n, YOLOv10n) in terms of parameter count, computational complexity, and detection accuracy. The results (Table 2) show that YOLOv8n achieves the optimal trade-off: it outperforms YOLOv5n and YOLOv10n by 4.7% and 1.9% in COCO mAP@0.5, while having only half the parameters and FLOPs of the slightly more accurate YOLOv7-tiny among the four models. Given the real-time processing requirements, YOLOv8n was selected as the base model for this study [17].

The YOLOv8 object detection network consists of four main parts: Backbone, Neck and Detection Head. The Backbone forms the foundation of the model, responsible for extracting features from the input image. These features constitute the basis for object detection in subsequent network layers. In YOLOv8, the backbone resembles CSP-Darknet. The Detection Head serves as the decision-making component, generating the final detection results. The Neck, positioned between the Backbone and Detection Head, is responsible for fusing and enhancing features.

The YOLOv8n loss function consists of three components: CIoU Loss for localization, which optimizes bounding box overlap, position, and shape matching; DFL for coordinate regression, which enhances bounding box accuracy via probabilistic modeling of coordinate distributions [18]; and for classification, it combines BCEWithLogitsLoss and Focal Loss, handling cross-entropy calculation while mitigating positive-negative sample imbalance. The loss function system selected for this study—comprising CIoU Loss, DFL, and the combination of BCEWithLogitsLoss and Focal Loss—is a proven and efficient scheme for small object detection in YOLO [19]. These components collectively deliver effective training gradients, thereby improving detection performance. Its specific structure is illustrated in Figure 3 [17,20].

#### 3.2.2. Model Training

This study used the YOLOv8n object detection algorithm (ultralytics version 8.3.174) implemented in the PyTorch deep learning framework (version 2.6.0, CUDA 12.6). After completing the environment configuration and installing the required dependencies, the default training parameters were adjusted to meet the needs of this research. The dataset was then fed into the convolutional neural network for model training. The training hyperparameters parameters were set as follows: 500 epochs, image size (imgsz) = 640, and batch size = 8. The remaining hyperparameters are shown in Table 3. Upon completion of the training, the model’s performance was evaluated using the established test set.

#### 3.2.3. Model Analysis

**Figure 4:**
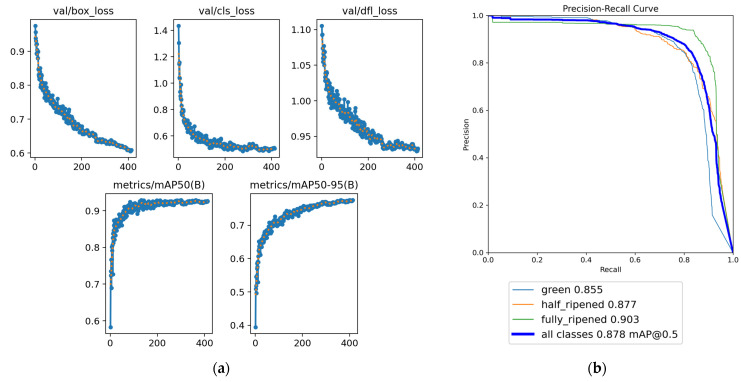
YOLOv8n model performance. (**a**) Training curves of YOLOv8n: loss decline and mAP rise; (**b**) Precision-Recall (PR) curve on the independent test set.

In this experiment, a YOLOv8n-based multi-category object detection model was constructed to recognize tomatoes at different maturity stages (immature, half-ripened, and fully ripened). The model was trained for 413 epochs before early stopping was triggered. As shown in the validation loss curves (Figure 4a), the box_loss and cls_loss decreased rapidly in the early training stage and gradually stabilized after approximately 200 epochs, converging to around 0.62 and 0.49 respectively at the best epoch (epoch 363). The training dfl_loss also showed a consistent downward trend and stabilized at approximately 0.93. Correspondingly, the mAP@0.5 and mAP@0.5:0.95 rose rapidly in the early stage and plateaued at their peak values of 0.929 and 0.775 respectively at epoch 363 (Figure 4a). This indicates that the model was thoroughly trained and possesses good generalization capability.

In terms of offline benchmark performance on the held-out test dataset, the overall precision rate reached 85.7%, and the recall rate reached 82.4%. The core metrics, mAP@0.5 and mAP@0.5:0.95, attained 87.8% and 72.7%, respectively (see Table 4). The Precision-Recall curves for each maturity category closely approximate the upper-right corner of the coordinate system (Figure 4b). Specifically, the mAP@0.5 values for immature (green), half-ripened (half_ripened), and fully ripened (fully_ripened) tomatoes were 85.5%, 87.8%, and 90.3%, respectively. This demonstrates the model’s excellent recognition precision and class differentiation capability for tomatoes at different maturity stages, effectively meeting the practical requirements of tomato maturity detection.

**Figure 5:**
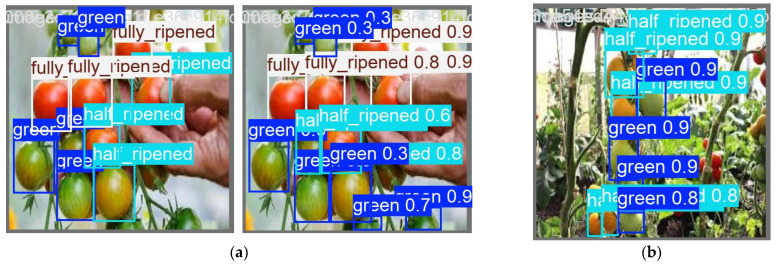
Sample tomato detection images. (**a**) Training processes; (**b**) Testing processes.

Sample tomato detection images from the training and testing processes are shown in Figure 5.

## 4. UWB Design and Application

### 4.1. UWB Working Principle

**Figure 6:**
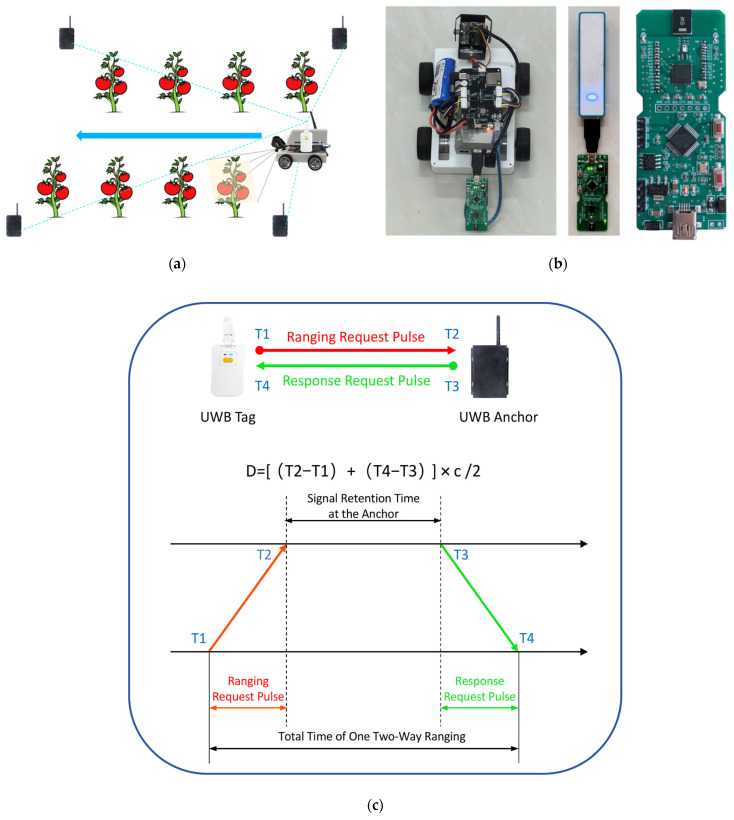

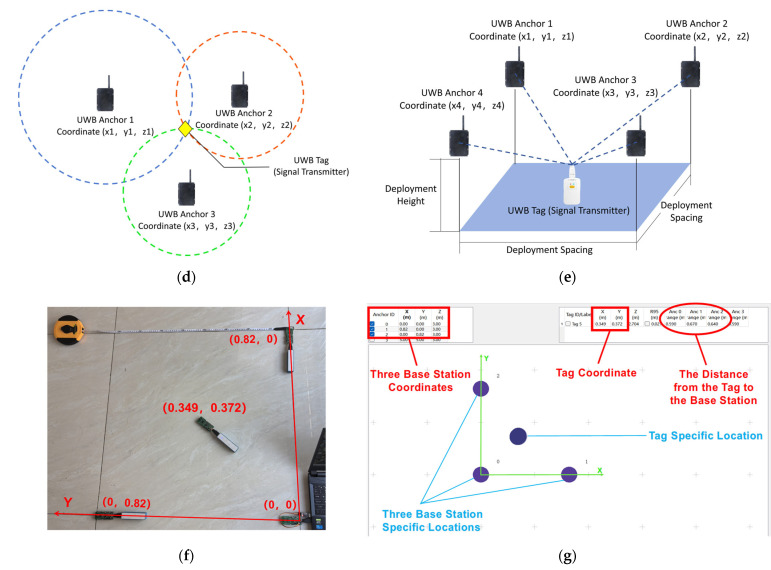
UWB module. (**a**) UWB-equipped robot workflow schematic; (**b**) Physical robot and BP-TWR-30 UWB hardware; (**c**) UWB positioning principle schematic; (**d**) Two-dimensional UWB positioning schematic; (**e**) Three-dimensional UWB positioning schematic; (**f**) Three-anchor UWB positioning setup; (**g**) Three-anchor UWB system interface screenshot.

Ultra-Wideband (UWB) is a novel wireless communication technology that transmits data by sending and receiving extremely narrow, nanosecond-level pulse signals, rather than relying on traditional sinusoidal carrier modulation. Its high time resolution makes it highly suitable for high-precision positioning. UWB can estimate location with sub-meter accuracy, provide real-time positioning at frequencies above 10 Hz, and locate devices up to 100 m away [21,22]. Furthermore, UWB offers advantages such as high precision, high security, low power consumption, and low cost [23]. Compared to GPS and BeiDou positioning, it does not require satellite signals, can achieve centimeter-level positioning within greenhouses, and offers strong resistance to multipath interference [24]. Even in enclosed, signal-occluded environments, UWB can still maintain sub-decimeter-level static positioning accuracy, which is unmatched by conventional satellite positioning technologies [25]. Among other common positioning technologies, WiFi offers low accuracy and Bluetooth suffers from poor stability [26], while UWB can reduce positioning errors by over 80%, meeting the requirements for fruit-level precision positioning of tomatoes. A schematic diagram of the inspection robot equipped with UWB is shown in Figure 6a. Figure 6b presents a photograph of the actual robot equipped with UWB, and the core hardware components of the BP-TWR-30 UWB positioning kit.

In this experiment, UWB ranging is based on the Two-Way Time of Flight method. Upon activation, the UWB module generates an independent timestamp. When the tag initiates a pulse signal at time T1 on its timestamp, the base station receives it at time T2. Subsequently, the base station sends a response pulse signal at time T3, and the tag receives this signal at time T4. T2 − T1 represents the transmission time of the ranging request pulse, and T4 − T3 represents the transmission time of the response request pulse. The distance between the two devices can be calculated using these two time differences and the speed of light, with the specific formula given as:

(1) $$D=\left[\left(T_{2}-T_{1})+(T_{4}-T_{3}\right)\right]\times c÷2$$

Refs. [21,27]. This principle is illustrated in Figure 6c.

UWB positioning typically uses three or four base stations. Each base station measures its real-time distance to the tag. Taking each base station as the center and the measured distance as the radius, circles are formed. The intersection point of these three (or four) circles represents the current position of the tag [28]. Two-dimensional (three anchor) and three-dimensional (four anchor) schematic diagrams of the UWB positioning principle are shown in Figure 6d,e. In practice, the spacing between base stations is typically kept within 50 cm to ensure high positioning accuracy. The geometric configuration of base stations directly determines the geometric dilution of precision of the positioning system, and unreasonable layout will significantly amplify ranging errors caused by non-line-of-sight conditions and multipath effects [25]. However, due to ranging errors, these three or four circles rarely intersect at a single point; instead, they intersect within a region, which constitutes the positioning error. The current system achieves a positioning accuracy within 24 cm, which is sufficient for locating tomatoes.

Figure 6f illustrates the setup for three-base-station UWB positioning. The computer side is connected to Base Station 0 via a serial port, and the base station’s coordinates are set to (0, 0). Base stations 1 and 2, connected to power sources, are placed 0.82 m from Base Station 0, with their coordinates set to (0.82, 0) and (0, 0.82), respectively. When the tag is placed within the triangle formed by the three base stations, the computer side displays its position on the coordinate graph. Additionally, a real-time information table in the upper right corner shows the distances from the tag to each base station and the tag’s coordinates. Thus, the tag’s position can be determined and used to locate the inspection robot, as shown in Figure 6g.

### 4.2. Localization Results

The intelligent tomato inspection robot, equipped with a UWB tag, conducted inspections in a tomato plantation with three pre-deployed UWB base stations. The real-time position of the robot was determined using the UWB system. During each inspection run, the robot generates and stores position data with continuous timestamps in the backend database in real time. Concurrently, it recorded continuous video footage of tomato plants. Post-inspection, we extracted frames from the recorded videos and recorded their corresponding timestamps. By matching these image timestamps with the synchronized positioning data from the backend database, the robot’s exact location at the moment each image was captured was accurately obtained. Subsequently, we fed these images into the trained YOLOv8n model to obtain detection results. By integrating the detection results with the corresponding positioning data, we determined the specific locations of tomatoes at different maturity stages. Finally, workers can locate each ripe tomato based on this spatial information, enabling timely harvesting and significant reduction in labor costs.

## 5. Experiments and Results

### 5.1. Experimental Setup

**Figure 7:**
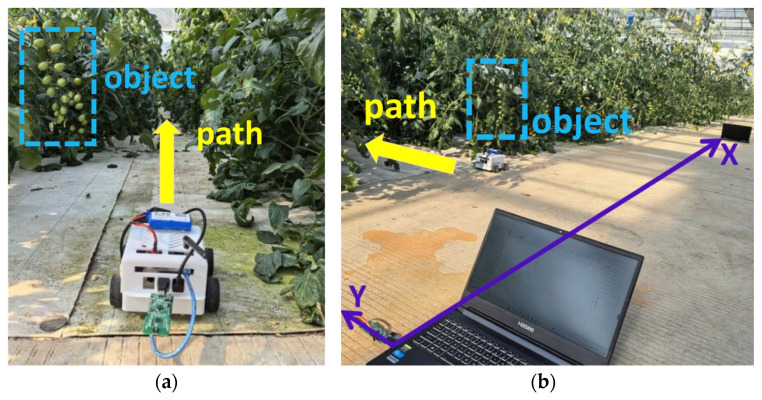
Robot inspection sample images. (**a**) Robot platform; (**b**) Complete system layout.

The test site for this project was the intelligent multi-span tomato planting greenhouse of Nanjing Qiaofeng Agricultural Development Co., Ltd. (Nanjing, China) On the test day, the air quality was good, sunlight was sufficient, and the temperature inside the greenhouse was approximately 20 °C. After initialization and pre-operation checks, the inspection robot collected data along the set route. Simultaneously, personnel manually recorded information such as the count of tomatoes at each maturity stage along the robot’s inspection route for subsequent comparison. We focused on operational stability, data accuracy, work efficiency, and energy consumption. Partial images during the robot inspection experiment are shown in Figure 7.

### 5.2. Results

#### 5.2.1. Overall Performance Evaluation

**Figure 8:**
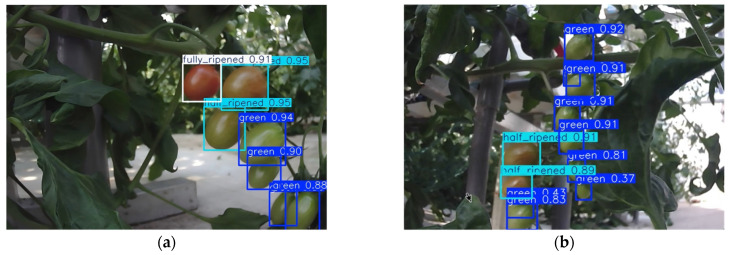
Field detection results of tomato ripeness. (**a**) Tomato clusters with mixed ripeness stages; (**b**) Tomato clusters with only half-ripened and immature tomatoes.

First, the robot captured images of tomatoes at three maturity stages. Then, these images were fed into the YOLOv8n model for detection and counting. As shown in Figure 8, the model successfully detected tomatoes at all three stages, with confidence scores all around 0.9—very close to 1. This demonstrates the model’s effectiveness in identification and detection.

Next, the robot was switched to inspection mode, with the 2-DOF camera gimbal set and fixed at an appropriate angle. Simultaneously, three UWB base stations were placed in an equilateral triangle configuration with 6 m spacing to conduct the inspection experiment. The robot was operated at approximately 0.1 m/s along the furrow. After the test, both the videos captured by the robot and those from the UWB positioning system were sampled at a frequency of one frame every 2 s. The extracted tomato images captured by the robot were then input into the YOLO model to obtain recognition results. Concurrently, these results were combined with the corresponding position information extracted from the videos of the UWB positioning system. The specific locations of tomatoes at each maturity stage were then determined.

**Figure 9:**
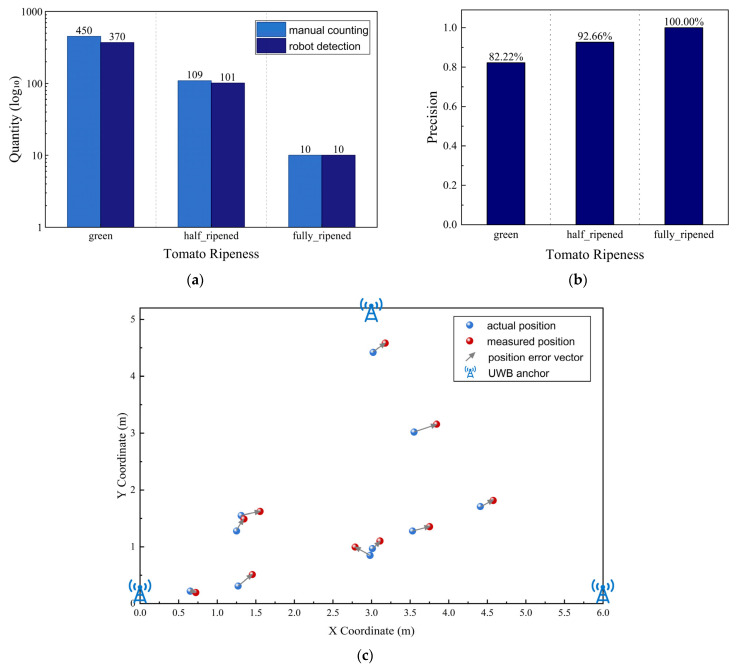
Experimental Results. (**a**) Number of tomatoes detected by the robot for each maturity stage; (**b**) Tomato ripeness detection precision; (**c**) The actual and measured positions of all ripe tomatoes.

During this robot inspection experiment, a total of 50 tomato clusters were selected. Manual counting yielded 450 immature tomatoes, 109 half-ripened tomatoes, and 10 fully ripened tomatoes. The inspection robot’s end-to-end field detection results identified 370 immature tomatoes, 101 half-ripened tomatoes, and 10 fully ripened tomatoes, resulting in recognition precision rates of 82.22% for immature tomatoes, 92.66% for half-ripened, and 100% for fully ripened, as shown in Figure 9a,b.

Notably, a measurable performance gap exists between the offline benchmark results in Section 3.2.3 and the end-to-end field detection results presented above. This gap represents a universal challenge in agricultural robot deployment, stemming primarily from environmental occlusion, background interference, and motion-induced image degradation. In real-world greenhouses, approximately 30% of tomatoes are partially or fully occluded by leaves, stems, and adjacent fruits. In contrast, our offline dataset contains only about 10% heavily occluded samples (defined as >50% of the fruit surface obscured), resulting in lower recall for occluded targets. Immature green tomatoes are most severely affected, as their color is nearly indistinguishable from background foliage, making partially occluded fruits prone to being missed. Furthermore, even at the optimal patrol speed of 0.1 m/s, minor motion blur and frame jitter are unavoidable during robot operation. These artifacts degrade image sharpness relative to the static images used for offline training and validation. As the core objective of this study is to detect and localize fully ripe tomatoes, this performance gap does not compromise the validity of our results, as we achieved 100% detection coverage for fully ripe fruits. Nevertheless, we will augment our dataset with more diverse field samples in future work to further improve the model’s overall recognition accuracy.

For context on these field results, we benchmark our model against two state-of-the-art lightweight tomato detectors. Meng et al.’s [5] improved YOLOv7-tiny achieved an mAP of 87.3% on the multi-ripeness cherry tomato detection task, while the YOLO-CT proposed by Qin et al. [4] attained an AP of 85.3% for ripe cherry tomato detection. By comparison, the YOLOv8n-based model proposed in this study achieved detection precisions of 82.22%, 92.66%, and 100% for immature, half-ripened, and fully ripe tomatoes, respectively, outperforming the aforementioned two cherry tomato-oriented lightweight models in overall detection accuracy, particularly in the precise identification of fully ripe tomatoes.

Simultaneously, the positions of the fully ripened tomatoes measured by the UWB positioning device were compared with their actual positions. For example, the actual position of the first ripe tomato was (0.65, 0.22), while the UWB positioning device measured it at (0.722, 0.197), yielding a localization error of approximately 0.076 m. A comprehensive comparison of all ripe tomato positions yielded a Root Mean Square Error (RMSE) of 0.185 m in the x-direction, 0.139 m in the y-direction, and an overall RMSE of 0.231 m for this experiment. In practical inspection operations, the row spacing of tomato plants in the plantation is approximately 0.8 m and the plant spacing is approximately 0.4 m. An error within 0.24 m does not affect the ability to locate the specific plant bearing a ripe tomato; workers can quickly identify ripe tomatoes based on this localization result, which meets the requirements of tomato inspection. The main sources of this error are the positive ranging bias caused by Non-Line-of-Sight (NLOS) propagation due to occlusion by plant branches and leaves in the greenhouse tomato environment, as well as the interference in first-path detection caused by multipath effects. In the future, we plan to optimize the base station layout using the Particle Swarm Optimization (PSO) algorithm and to suppress the bias and multipath interference by combining NLOS confidence weighting with the UWB-Inertial Measurement Unit (IMU) tightly coupled filtering [29]. Figure 9c shows the actual and measured positions of all ripe tomatoes in this experiment.

Overall, the intelligent inspection robot demonstrated high operational stability (no malfunctions or stalling during the inspection process), relatively high data accuracy (high image recognition rates, low positioning errors), high work efficiency (capable of inspecting a 50 m long tomato furrow in 10 min (±1 min)), and low energy consumption (a continuous operational battery life of 2 h (±10 min)).

#### 5.2.2. Parameters Analysis

The distance parameter directly affects the effectiveness of feature extraction from crop targets. Excessive or insufficient shooting distance can lead to blurred target features or information loss, thereby significantly reducing the recognition precision of deep learning models. Optimizing acquisition parameters is crucial for improving field detection performance [30]. During the experiment, we set variables including the distance between the robot’s camera and the tomato plant base, the robot’s patrol speed, and the elevation angle of the robot’s camera gimbal. The optimal parameters for the inspection robot’s operation were ultimately determined as follows: a camera distance of approximately 20 cm from the tomato plant base, a patrol speed of approximately 0.1 m/s, and a camera gimbal elevation angle of approximately 20°. Furthermore, we analyzed the base station geometry and the robot movement direction to evaluate their effects on UWB positioning accuracy.

- Camera Distance

**Figure 10:**
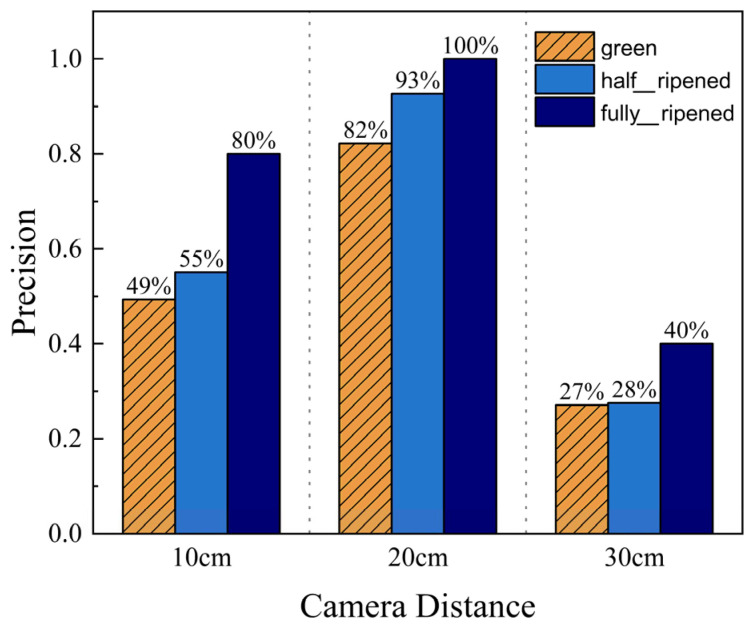
Ripeness detection precision at different camera distances.

When the robot’s camera was positioned too close to the tomato plant base, it often failed to capture all tomatoes in the row, and some leaves obstructed its field of view. At a camera distance of approximately 10 cm, the recognition precision was only 49.33% for immature tomatoes, 55.05% for half-ripened, and 80% for fully ripened. When the robot’s camera was positioned too far from the tomato plant base, the captured tomato images appeared blurred or too small. Under such conditions, the YOLO model could not accurately identify all tomatoes within its field of view. At a camera distance of approximately 30 cm, the YOLO model recognized only about 30% of the tomatoes within its field of view. In this case, the recognition precision was 27.11% for immature tomatoes, 27.52% for half-ripened, and 40% for fully ripened. The recognition precision under this variable condition is presented in Figure 10. This variable does not influence the positioning accuracy.

- Patrol Speed

**Figure 11:**
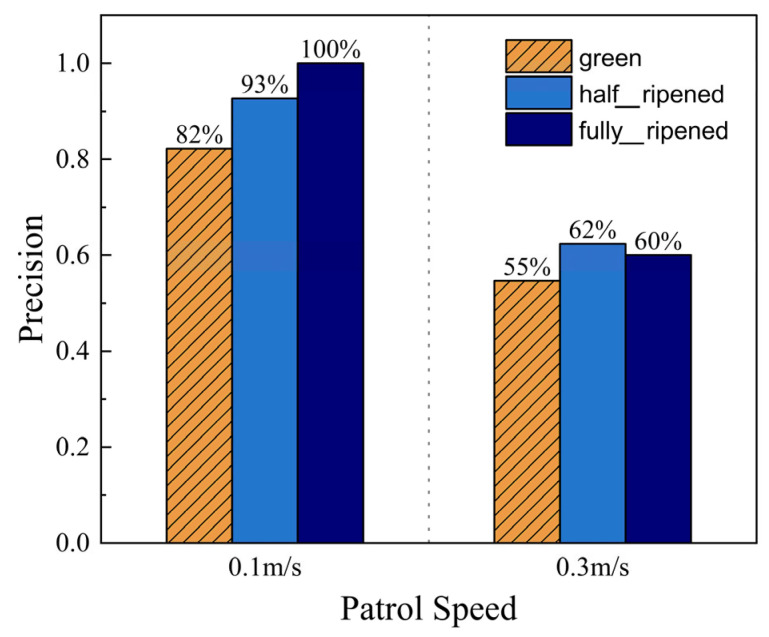
Ripeness detection precision at different patrol speeds.

In experiments on robot patrol speed, we found that a speed of 0.1 m/s was most suitable. If the speed was too high, the tomato images captured by the robot became blurred, preventing accurate recognition by the YOLO model. In the comparative experiments, the robot’s patrol speed was set to 0.3 m/s. The recognition precision was 54.67% for immature tomatoes, 62.38% for half-ripened, and 60% for fully ripened. The recognition precision under this variable condition is presented in Figure 11. This variable does not influence the positioning accuracy.

- Camera Gimbal Elevation Angle

**Figure 12:**
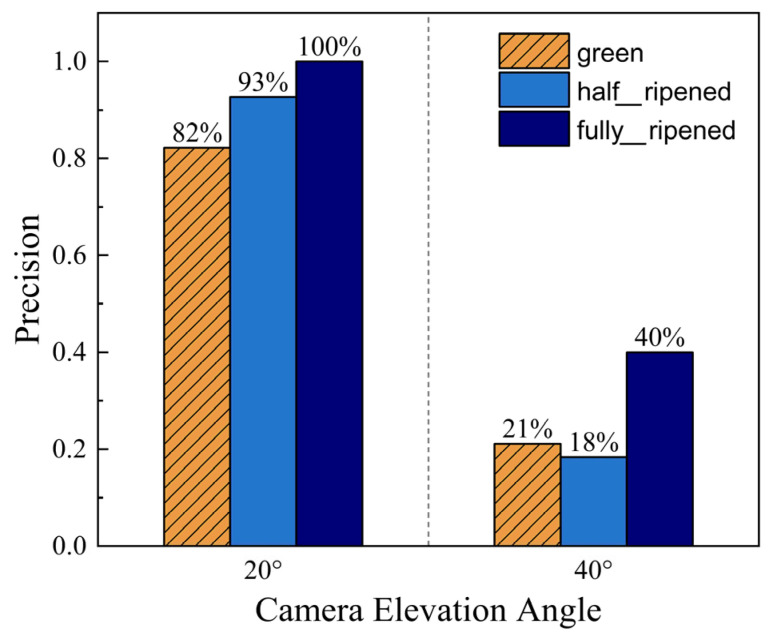
Ripeness detection precision at different camera elevation angles.

Furthermore, the elevation angle of the robot’s camera gimbal should not be too large, with 20° being optimal. If the elevation angle was too large, the camera would capture the greenhouse roof along with the bright sky above it, resulting in overexposed images that could not be recognized. In the comparative experiments, the robot’s camera gimbal elevation angle was set to 40°. The recognition precision was 21.11% for immature tomatoes, 18.35% for half-ripened, and 40% for fully ripened. The recognition precision under this variable condition is presented in Figure 12. This variable does not influence the positioning accuracy.

- Base Station Geometry and Robot Movement Direction

**Figure 13:**
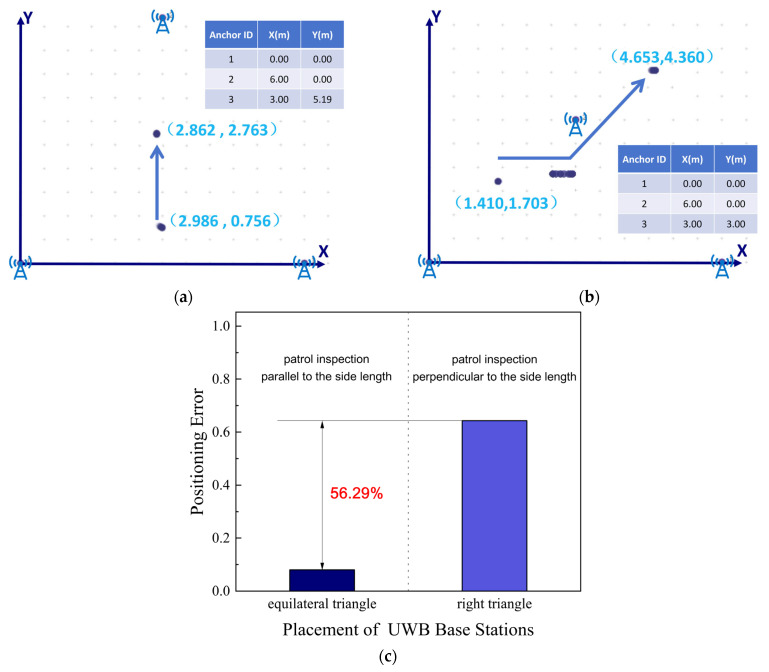
UWB positioning accuracy under different base station geometries and robot movement directions. (**a**) Equilateral triangle layout with perpendicular movement; (**b**) Right triangle layout with parallel movement; (**c**) Comparison of positioning errors. In the figures, blue icons denote base stations (identical to those in Figure 9c), blue dots represent the specific positions of the robot localized by the system, and blue arrows illustrate the robot’s positioning trajectory.

In unstructured field environments, the compatibility between the placement configuration of the UWB base stations and the robot’s direction of movement directly affects positioning accuracy [31]. An equilateral triangle layout provides more uniform signal coverage, reducing errors caused by occlusion and multipath interference. We set the placement configuration of UWB base stations as a variable, positioning the three base stations in right triangle and equilateral triangle configurations respectively. Subsequently, we had the robot move respectively along directions parallel and perpendicular to the triangle sides within each of the two above base station configurations. Experimental results revealed that when the robot moved perpendicular to the sides during inspection within the three base stations arranged in an equilateral triangle, it achieved the highest positioning accuracy, with an error of 8% (less than 10%). When the robot moved parallel to the sides within the three base stations arranged in a right triangle, the positioning error was largest. In extreme cases, the robot’s positioning result even deviated outside the coverage area of the three base stations (see Figure 13b), with a positioning error of 64.29%. The positioning results of the two experiments are presented in Figure 13a,b, with Figure 13c illustrating the comparison of their positioning errors.

### 5.3. Discussion

In this experiment, we set five variables and conducted multiple inspection tests on tomatoes within the selected area. During the process, extreme situations occurred, such as leaf occlusion, image overexposure, and UWB positioning deviations. However, after multiple variable adjustments—with the robot’s camera positioned approximately 20 cm from the tomato plant base, a patrol speed of approximately 0.1 m/s, a camera gimbal elevation angle of approximately 20°, and the robot moving perpendicular to the sides within the three base stations arranged in an equilateral triangle—the recognition precision reached 82.22% for immature tomatoes, 92.66% for half-ripened tomatoes, and 100% for fully ripened tomatoes. Under these parameters, the tomato intelligent inspection robot can effectively address various unexpected and extreme situations, accurately and efficiently identify and locate ripe tomatoes, and promptly alert workers for harvesting, thereby realizing unmanned inspection functionality in tomato plantations.

Although existing work has achieved promising results, the proposed tomato intelligent inspection robot still has several aspects that require further improvement. These primarily include poor detection performance for multiple overlapping targets and for small targets at a distance within the field of view. Such limitations mainly occur in scenarios involving leaf occlusion or when the target is similar in color to the background. Our next objectives are to further expand the dataset by adding more images of tomatoes occluded by leaves and images with complex backgrounds. We also plan to increase the camera resolution and meanwhile adopt the Wise-IoU loss function to replace the standard CIoU loss function. Wise-IoU can reduce excessive interference from geometric factors through a dynamic weighting and outlier evaluation mechanism, allowing the model to focus more on overlapping regions of ordinary quality that are difficult to regress, thereby improving detection performance in dense scenarios [32]. In addition, we consider adding a small-object detection layer (P2 head) to enhance the detection of small, distant tomatoes within the field of view. Similar P2-based architectural enhancements have been shown to significantly improve small object detection accuracy in agricultural contexts, as demonstrated for green crisp plum detection in complex orchard environments [33].

## 6. Conclusions

This study designed and implemented an intelligent tomato inspection robot that integrates ripeness recognition and precise localization. The core innovation of this work lies in the system-level integration and optimization of perception, positioning, control, and mobility modules for greenhouse tomato inspection scenarios. We focused on solving the practical challenges of deploying intelligent systems in real agricultural environments, including low cost, high reliability, and minimal infrastructure requirements. By adopting a layered modular architecture, the proposed system flexibly adapts to complex field environments, providing an efficient solution for the intelligent management and timely harvesting of tomato crops. The main conclusions of this study are as follows:

1. A layered, modular robot platform using a Raspberry Pi 5 as the main controller was developed. The robot weighs only 0.55 kg, with dimensions of 34 cm × 18 cm × 14 cm. This lightweight and compact design not only significantly reduces hardware costs but also enables the robot to flexibly navigate through narrow tomato ridges, achieving autonomous movement without relying on fixed tracks.
2. A multi-dimensional tomato ripeness image dataset (categorized into immature, half-ripened, and fully ripened) was constructed, and a lightweight object detection model based on YOLOv8n was successfully trained. After 413 epochs of training, the model achieved an overall mAP@0.5 of 87.8% and an mAP@0.5:0.95 of 72.7% on the held-out test dataset. In field tests, it achieved precisions of 82.22%, 92.66%, and 100% for immature, half-ripened, and fully ripe tomatoes, meeting practical inspection requirements. Notably, the recognition precision for fully ripe tomatoes reached 100%, demonstrating excellent phenotypic detection capabilities.
3. To overcome the susceptibility of traditional navigation methods to occlusions in greenhouse environments, an Ultra-Wideband (UWB) positioning system with strong anti-interference capabilities was introduced. The overall Root Mean Square Error (RMSE) of the system is only 0.231 m, and the positioning error is stably maintained within 0.24 m, fully satisfying the requirements for fruit-level high-precision localization during inspection tasks.
4. Through multiple completed field tests, we have identified the optimal parameters for robot inspection performance: a camera distance of approximately 20 cm from the tomato plant base, a patrol speed of approximately 0.1 m/s, a camera gimbal elevation angle of approximately 20°, and the robot moving perpendicular to the sides within the three base stations arranged in an equilateral triangle. Under these parameters, both the recognition precision for tomatoes at the three maturity stages and the positioning accuracy of the UWB system achieve optimal performance. Under optimal parameter configurations, the robot can efficiently complete the inspection of one side of a 50-m planting ridge within 10 min (±1 min). Furthermore, the device features a continuous operational endurance of up to 2 h (±10 min). It effectively resolves the problems of inefficient manual inspection and delayed harvesting, demonstrating significant value for practical application and promotion.

Despite these promising results, this study has several limitations that warrant further investigation:

1. Detection performance limitations: The model’s precision degrades significantly under conditions of severe leaf occlusion and for densely overlapping targets. In real-world field conditions, the recognition precision for immature tomatoes is markedly lower than that for fully ripened tomatoes.
2. Positioning performance limitations: The UWB system exhibits elevated error levels at the edges of base station coverage areas, and positioning accuracy decreases slightly during dynamic robot movement.
3. Functional limitations: The system is currently limited to tomato ripeness detection and lacks the ability to detect plant diseases, pests, or growth abnormalities. Additionally, the robot operates along pre-programmed routes and lacks autonomous obstacle avoidance functionality.

To systematically address the aforementioned limitations and further enhance the real-world applicability of the proposed system, our future work is structured into three phases as follows:

- Targeted system enhancements

First and foremost, we will systematically optimize the intelligent tomato inspection robot. Specifically, based on the experimental findings and practical application requirements of this study, we will enhance the stability and accuracy of the camera and UWB sensors through targeted hardware refinements, improve the YOLOv8n model’s generalization and inference speed by augmenting the dataset with heavily occluded tomato samples and replacing the CIoU loss function with the Wise-IoU loss function, and enhance overall system performance to boost the robot’s adaptability and robustness across diverse agricultural environments and planting scenarios.

- Medium-term functional expansions

Moving to the medium term, we will focus on expanding the robot’s core practical functionalities. Specifically, we plan to integrate autonomous decision-making capabilities and draw upon the technical approach of UWB-IMU integrated navigation [29] to assess road conditions based on field data and autonomously plan routes for inspection tasks. Furthermore, we will extend the YOLOv8n model for intelligent detection of common tomato diseases using phenotypic data such as leaf color and fruit skin condition. As deep learning technologies have been proven capable of achieving precise disease detection through the extraction and analysis of crop phenotypic features [34,35], this capability will enable timely alerts to workers for targeted treatment.

- Long-term strategic developments

For long-term strategic development, we will explore broader application scenarios and system-level integration. We aim to extend the robot’s application to other dwarf crops such as eggplant and pepper, which will require adapting its detection framework to recognize different crop varieties and their respective maturity stages. Additionally, we will develop interoperable interfaces with agricultural production management systems for real-time data sharing and analysis, thereby expanding the robot’s application scope in precision agriculture [36]. Finally, as a forward-looking research direction, we will explore the feasibility of adding a planting strategy optimization module that can provide personalized cultivation recommendations based on tomato growth performance under different agronomic practices.

## Figures and Tables

**Table 1:** Annotated list of components for the tomato intelligent inspection robot.

Main Component	Model	Performance Parameters	Selection Justification
Host Computer	Raspberry Pi 5	4-core 2.4 GHz, 8 GB LPDDR4X	Provides sufficient computing power with low cost and high integration [3].
Cooler	Active Cooler (PWM Fan with Titanium Alloy Heat Dissipation Base & Thermal Pad)	5 V DC PWM fan, 1.09 CFM, 8000 RPM	Maintains Pi 5 at 42–80 °C to prevent throttling in 25–35 °C greenhouse.
Expansion Board (Lower Computer)	ESP32-S3	Dual-core 240 MHz, 16 MB Flash	Offers rich interface expansion and high integration [3].
2-DOF Camera Gimbal	Camera + Servo Motor	2 MP, 120° horizontal FOV	Adjusts angle to avoid leaf occlusion; high frame rate ensures motion clarity.
Power Supply	7.4 V Lithium Battery	2000 mAh Lithium battery pack	Features lightweight design; supports 2 h continuous operation.
Wheels	Rubber Tires	48.0 × 21.5 mm	Provides four-point support and free steering.
Chassis	ABS Engineering Plastic	31.0 × 15.0 × 3.1 cm	Serves as base support.
Housing	ABS Engineering Plastic	15.1 × 11.5 × 0.5 cm	Offers protective structure.
Antenna	WiFi Antenna	2.4/5 GHz dual-band, 867 Mbps, 3 dBi gain	Improves greenhouse signal stability and supports remote debugging.

**Table 2:** Performance comparison of lightweight YOLO models.

Model	Parameters (M)	FLOPs (G)	COCO mAP@0.5
YOLOv5n	1.9	4.5	0.457
YOLOv10n	2.3	6.8	0.485
YOLOv8n	3.2	8.7	0.504
YOLOv7-tiny	6.2	13.8	0.529

Data source: Ultralytics official benchmark (640 × 640 input).

**Table 3:** Training Hyperparameter Configurations of the Detection Model.

Training Hyperparameters	Parameter	Value
Learning Rate Schedule	lr0	0.00125
	lrf	0.01
	LR Scheduler	Linear Decay
Optimizer Configuration	Optimizer	SGD
	Momentum	0.937
	Weight Decay	0.0005
Loss Function Weighting	Box Loss Weight	7.5
	Cls Loss Weight	0.5
	DFL Loss Weight	1.5
Data Augmentation Strategy	Mosaic Augmentation	Probability: 1.0
	Horizontal Flip	Probability: 0.5
	HSV Color Augmentation	H: 0.015, S: 0.7, V: 0.4
	Random Erasing	Probability: 0.4
	Auto Augmentation	RandAugment
Early Stopping Policy	Patience	50

**Table 4:** Offline benchmark recognition performance statistics of the YOLOv8n model on the test dataset.

Class	Precision	Recall	mAP@0.5	mAP@0.5:0.95
All	0.857	0.824	0.878	0.727
green	0.857	0.785	0.855	0.672
half_ripened	0.844	0.804	0.877	0.727
fully_ripened	0.869	0.881	0.903	0.782

## Data Availability

The data that support the findings of this study are available from the corresponding author upon reasonable request.
